# Supplementary material for: Complexome profiling of the Chlamydomonas psb28 mutant reveals TEF5 as an early PSII assembly factor
Source: Plant Cell. 2025 Mar 17;37(6):koaf055. doi: 10.1093/plcell/koaf055 (PMC12164590; doi:10.1093/plcell/koaf055)
Supplement: koaf055_Supplementary_Data [file koaf055_supplementary_data.zip › psb28_interactive.zip!/plots/intensity/qualityPlots/peptides/_Cre14.g611950.t1.1.html]

### Cre14.g611950.t1.1

Trivial name:   
SeqCoverage [median]: 29.50  
Mass: 64.22 kDa  
  
 

| Sequence | MissedCleavage | Mass [Da] | wtIntensity | muIntensity | Score |
| --- | --- | --- | --- | --- | --- |
| AAADAPSDAGR | 0 | 1000.46 | 2978.93 | 479.14 | 119.68 |
| AAAVLTESAEDVR | 0 | 1330.67 | 2563.90 | 2024.52 | 138.66 |
| AFVSSPALVR | 0 | 1045.59 | 2694.42 | 1846.80 | 85.21 |
| AQAEARDEVER | 1 | 1272.61 | 1623.01 | 1663.79 | 90.91 |
| GSEEVGFGK | 0 | 908.42 | 1880.56 | 700.41 | 86.79 |
| KPGVAAIHEFVLQAPDSTSR | 1 | 2122.12 | 0.00 | 3923.75 | 72.42 |
| KVELLDGYSR | 1 | 1178.63 | 784.65 | 644.96 | 68.54 |
| LAAATTAEDVADALSEEVPSGLREEDAR | 1 | 2885.39 | 422.06 | 0.00 | 67.36 |
| LTVVSSPVR | 0 | 956.57 | 5273.39 | 4048.23 | 90.66 |
| QQLLGQYNTLAGK | 0 | 1432.77 | 0.00 | 1795.38 | 99.81 |
| TVADGYSR | 0 | 867.41 | 333.97 | 0.00 | 58.98 |
| VGTENATVPAQVGER | 0 | 1526.77 | 2793.71 | 2882.83 | 115.14 |
| VVTSPLAPDRPLTVVQPQEGAK | 1 | 2301.27 | 5243.77 | 8440.19 | 88.26 |
|  |  |  | 26592.4 | 28450.0 |  |
